# Cannabis and Myocardial Infarction: Risk Factors and Pathogenetic Insights

**Published:** 2017-07-22

**Authors:** Justin Lee, Navneet Sharma, Farnam Kazi, Irini Youssef, Alyson Myers, Jonathan D. Marmur, Moro O. Salifu, Samy I. McFarlane

**Affiliations:** State University of New York-Downstate, Department of Medicine, Divisions of Cardiovascular Medicine and Endocrinology, Brooklyn, NY 11203, USA

**Keywords:** Cannabis, Marijuana, Myocardial Infarction, Metabolic derangement, Inflammation, Cardiovascular, Platelet

## Abstract

Cannabis use in the US is rising with increased legalization. It has been noted that there is a five-fold increase risk of Myocardial Infarctions (MI) in the first hour after cannabis use. Traditional risk factors for MI include diabetes, hypertension and dyslipidemia. The rising use of cannabis may have ushered in an additional MI risk factor to be added to the list; that is cannabis. In this review, we discuss the growing use of cannabis and potential link with MI, highlighting the common pathogenic hypotheses linking these risk factors.

## Introduction

As of January 1, 2017, the use of both recreational and medical cannabis has been legalized in 8 states (Alaska, California, Colorado, Maine, Massachusetts, Nevada, Oregon and Washington) and the District of Columbia in the United States [1]. With the increasing legalization of the drug, the use of cannabis has also been on the rise. Additionally, a recent study has suggested that longer-term moderate/heavy cannabis use during early and late 20s is associated with negative health outcomes by age 50 [2]. It is important to understand the possible negative health effects of cannabis use, because the youth population carries on a large portion of recreational use of the drug. With the rising use and demand for cannabis, commercial preparations containing synthetic cannabinoids have also rapidly emerged. Although synthetic alternatives are often assumed to be safe and legal alternative to cannabis, their enhanced metabolic effects and potential toxicity warrant further investigation into their safety [3]. Moreover, current evidence on the effect of cannabis use on health outcomes is limited. Given the rapid progressive movement towards legalization of cannabis recently and paucity of data on potential harms of cannabis use, it remains an active area of research.

Cardiovascular disease (CVD) is the number one cause of death in the United States with 1 in every 4 deaths attributed to CVD [4]. It includes Coronary Artery Disease (CAD), including Myocardial Infarction (MI), hypertension, Congestive Heart Failure (CHF), arrhythmias, Peripheral Vascular Disease (PVD) and strokes. A majority (70%) of CVD risk is attributable to modifiable risk factors, such as smoking [5]. It is also interesting to note that cannabis use was significantly higher amongst black Caribbean adolescents, compared to other ethnicities in a study conducted in an urban setting in UK [6]. The effect of cannabis use on CVD and as potential risk factor for MI is largely unknown.

In this review, we will explore the potential mechanisms that lead to myocardial infarctions associated with cannabis use.

## Metabolic Derangement

Endocannabinoids and their endogenous receptors (CB_1_R and CB_2_R) play an important role in regulating the body’s energy balance, insulin sensitivity and lipid metabolism [7, 8]. It is known that the effect of D9-Tetrahydrocannabinol (THC), the primary psychoactive component of *Cannabis sativa*, acts on its receptors is associated with an acute increase in appetite and high caloric intake [9]. High caloric intake and polyphagia associated with cannabinoid consumption or inhalation can lead to obesity and its metabolic consequences, such as diabetes mellitus, cardiovascular diseases, sleep disorders and chronic kidney disease. In addition to the metabolic derangement precipitated by obesity, cannabinoids activate CB_1_ receptors in peripheral tissues causing lipogenesis, reduced insulin responsiveness and impaired secretion of insulin in adipose tissues, liver, skeletal muscles and pancreas [10]. The culmination of such derangements is known to accelerate endothelial dysfunction. Multiple studies have shown that acute, short-term activation of CB_1_ is associated with rapid rise in appetite, food intake and body weight as well as glucose intolerance in young, healthy males [10–12]. Whether these metabolic effects have causal relationship or are independent from each other is still not well understood. Nonetheless, there have been animal studies that have shown chronic CB_1_ stimulation by endocannabinoids leads to adiposity independent of its effects on appetite provocation [10]. It is important to recognize and understand the association between the use of THC and its metabolic consequences, because obesity, along with diabetes mellitus are considered as a major risk factor and risk factor equivalent for CVD.

Chronicity of cannabis use also appears to have its effects on lipid and carbohydrate metabolism in human body. In the Coronary Artery Risk Development in Young Adults (CARDIA) study, subjects with more extensive use of cannabis was associated with higher caloric intake [13]. Moreover, the increase in caloric intake was hypothesized to be closely linked with the concomitant increase in alcohol consumption [13]. Activation of CB_1_ is widely thought to be the culprit for such polyphagic behavior through the stimulation of plasma ghrelin and leptin [14, 15]. It is interesting to note that prevalence of obesity was lower in chronic cannabis users compared to non-users [16]. Furthermore, higher frequency of cannabis use was significantly associated with lower abdominal fat content and BMI, from the NHANES III study [16]. Effect of chronic cannabis use on insulin secretion was investigated by Muniyappa et al using C-peptide deconvolution and oral glucose tolerance test modeling, total insulin secretion, β-cell glucose sensitivity, rate sensitivity, and potentiation of insulin secretion. From the study results, chronic cannabis smoking does not appear to affect glucose sensitivity in peripheral tissues and pancreatic β-cell function, leading to normal glucose tolerance in long-term heavy users [10]. These findings are consistent with the results of the aforementioned CARDIA and NHANES III studies. It is important to note that the hypotheses proposed by past, historical studies regarding short-term activation of CB_1_ causing glucose intolerance are quite contrary to these new findings [10, 12]. It is thus possible to deduce that the effect of cannabinoids on its CB_1_ receptors differ with chronicity. Similarly, a case-crossover study conducted also suggests potential danger of short-term activation of CB_1_ receptor from cannabis smoking in patients with acute myocardial infraction (MI) as opposed to chronic use, as the risk of MI was elevated almost 5-folds in the hour after smoking and subsequently declined rapidly after the initial hour [17].

Results from CARDIA and NHANES III studies suggesting the association between cannabis use and lower BMI with lower abdominal fat content are interesting to note, as it is counterintuitive to believe that higher caloric intake would result in such reduction in body mass and fat deposition, or the lack there of [13, 16]. However, upon further investigation of relative amount of abdominal fat distribution, a study conducted by Muniyappa et al. has found that cannabis smokers had significantly higher visceral fat content, as opposed to subcutaneous fat, when compared with non-users [10]. This finding is significant because the ratio between visceral adipose tissue and subcutaneous adipose tissue is an independent predictor of cardiovascular events, irrespective of presence of risk factors [18]. Higher visceral adipose tissue content leads to higher incidence of obesity-associated metabolic dysregulation, involving multiple endocrine, metabolic and immunologic functions, when compared to subcutaneous adipose tissues [19]. Visceral adiposity is also strongly associated with long term alteration of cardiac structures in hypertensive and ischemic cardiomyopathy [20]. From the data suggested by recent studies, both body fat distribution and adipocyte phenotype are stronger determinants for fatal cardiovascular outcomes in obese patients than increased general adiposity. Higher visceral adiposity can be a significant link that leads cannabis users to multiple metabolic derangement and ultimately into fatal cardiovascular events.

## Inflammation

The cannabinoid receptors are part of the G-protein coupled receptor family that propagate downstream signaling at the cellular level upon their activation by respective ligands [17]. Based on pre-clinical studies, CB_1_ receptor activation leads to inflammation and atherosclerotic effect, while CB_2_ receptors mediate anti-inflammation and immuno suppression [21–23]. Consequence of such immunomodulation by cannabinoid is an area of ongoing research. However, extensive reviews have outlined some of the downstream effects of CB receptor activation that potentially leads to systemic pro-inflammatory state as well [24]. It has been suggested that cannabinoid-induced activation of G-protein coupled receptor 55 (GPR55) results in increased IL-12 and TNF-α, which in turn raises endocytic activity in monocytes, potentially leading to foam cell formation and atherosclerosis [24].

It is interesting to note that cannabidiol (CBD), an analog of THC has quite the contrasting effect on the body, compared to its counterpart. In a mouse model from recent study, it has shown that CBD inhibited macrophage’s production of TNF-α from lipopolysaccharide chains [25]. In addition, CBD has shown its effectiveness in reducing other pro-inflammatory cytokines, such as NO and IL-β in another mouse model with Alzheimer’s disease related neuroinflammation [26]. These antagonistic properties of CBD on inflammation has made it an ideal target for pharmaceutical a agent, especially given that it has less psychoactive profile compared to THC. It is likely that these anti-inflammatory actions of CBD are modulated by the CB_2_ receptor as opposed to CB_1_receptors, however the complete biochemical pathway still needs to be elucidated.

Although multiple studies have shown the potential anti-inflammatory role for one cannabinoid analog, others have suggested cannabinoid’s action on promoting the release of pro-inflammatory cytokines, leading to contrasting hypotheses. A recent NIH study used C-reactive protein (CRP) as a marker for inflammation, showing decreased CRP levels in cannabinoid users [27]. The Justification for the Use of Statins in Prevention: an Intervention Trial Evaluating Rosuvastatin (JUPITER) trial highlighted the importance of CRP reduction with statin use in patients with normal metabolic profiles. Statin use in those with elevated CRP, but normal LDL, showed reduction in fatal and non-fatal MIs. Further, those on a statin also had reduced need for coronary interventions. Therefore, it remains to be seen whether those with CRP elevation resulting from cannabis use can benefit similarly from statin use [28, 29].

It has also suggested that these immunomodulation and reduced cytokine production can lead to altered immune response, higher susceptibility for infections and overall less favorable outcome amongst cannabis users [27]. With further investigation, understanding the complexity of cannabinoid’s inflammatory effect on the body can provide additional risk factor for cardiovascular diseases and future screening tools in serum markers for population of ever-increasing cannabis users in our society.

## Effects of THC on Cardiovascular System

While Metabolic and Inflammatory effects accrue over time with cannabis use, cardiovascular effects from cannabis use can precipitate to a MI much more acutely. Myocardial effects result from alterations in coronary blood flow and heart rate promoting myocardial ischemia and potential infarction [30]. Several studies have reported that acute THC use may be involved in reducing coronary blood flow. Additionally, intravascular ultrasounds on patients experiencing THC associated myocardial infarction usually find no evidence of atherosclerotic CAD. Coronary angiography usually confirms coronary vasospasm and platelet thrombus formation without underlying atherosclerosis [31]. Moreover, myocardial oxygen supply is further restricted by an increased concentration of carboxyhemoglobin leading to a reduction in oxygen carrying capacity of red blood cells [17]. Further exacerbating the myocardial oxygen supply are elevations in both heart rate and blood pressure, resulting in reduction of diastolic coronary filling and elevated diastolic coronary pressures. Therefore, the reduction in coronary blood flow in combination with reduced oxygen carrying capacity and potential systemic and coronary vasoconstriction lead to an increase in myocardial oxygen supply-demand mismatch, resulting in ischemia [17]. Patients suffering from preexisting cardiac conditions such as stable angina are especially prone to develop symptoms. Decreased exercise time to angina was reported in volunteers who use THC [32]. Other studies have proposed that the hemodynamic response caused by THC interrupts perfusion in susceptible atherosclerotic plaques. The role of beta blockers in reducing the myocardial oxygen supply-demand mismatch in cannabis users warrants further investigation.

The effects of THC on the peripheral vasculature results from two main effects namely: vasoactive effects and inflammatory effects. Vasoactive alterations resulting in changes in blood pressure are use dependent basis. Furthermore, in certain situations vasoactive changes can overlap with inflammatory changes and may be indistinguishable as is the case in thromboangiitis obliterans induced by THC, also known as cannabis arteritis [30]. It was demonstrated that THC binds the PPARγ receptor leading to short term increase in superoxide dismutase activity and bioavailability of nitric oxide, a potent vasodilator [33]. Conversely, long term THC use has been associated with increased peripheral vascular resistance [34]. The proposed mechanism resulting in vasoconstriction with chronic THC exposure is related closely to increase in vasoconstrictive prostanoids and antagoinism of endogenous vasorelaxing endocannabinoid anandamide [33–36]. Vasoconstriction may also be affected by the properties of the vessel itself. In vessels where the predominant vasodilating factor is Endothelium-derived Hyperpolarizing Factor, THC inhibits vasodilation and promotes vasoconstriction. Further, it should be noted that alterations in sympathetic tone may play a role in variable vasoactive effects of THC [37]. THC mediated vasoconstriction was inhibited in the rabbit ear artery with denervation and alpha adrenergic blockade, underscoring the potential sympathetic effects of THC. Chronic use leading to vasoconstriction along with prolonged inflammation can manifest as claudication, Raynaud’s phenomenon and ischemic digital ulcers. This disease can be difficult to distinguish from effects of smoking tobacco given the fact that the both substances are often used simultaneously. However, studies have shown that THC exposure led to an earlier presentation of thromboangiitis obliterans in individuals who abused both [30]. The role of calcium channel blockers in reducing peripheral vasculature burden in cannabis user needs further exploration.

## Effects of THC on Platelets

Presence of CB_1_ and CB_2_ receptors on platelet surface has been identified as a potential target for activation of platelet aggregation. Cannabinoids have been found to induce significant amount of arachidonic acid production in human platelets [38]. In ram and sheep models, THC potentiates cyclooxygenase 1 and 2 (COX-1, COX-2) leading to thromboxane A2 and subsequent prostaglandin production [39]. These findings are significant as both arachidonic acid and COX are proinflammatory molecules that can lead to endothelial injury, platelet activation, vasoconstriction and subsequent rise in the risk of cardiovascular event.

THC also induces CB_1_ and CB_2_activation that has been shown to activate platelet aggregation via increased platelet Glycoprotein IIa/IIIb and P-selectin expression [40, 41]. Additionally, THC associated increase in inflammation leads to production of 2-Arachidonoylglycerol (2-AG) that serves as a precursor Arachidonic Acid [40]. Initial effects of 2-AG on platelet aggregation begin with Phosphotidylinositol 3 Kinase/AKT pathway leading to myosin light chain kinase phosphorylation and subsequent actin polymerization that result in conformational changes in platelet structure. Additionally, the conformation changes result in ATP secretion and 2-AG mediated platelet aggregation [42]. One study investigating 2-AG mediated platelet aggregation showed retardation in platelet aggregation in subjects being treated with aspirin and/or Plavix. The same study also showed subjects on Aspirin and/or Plavix had minimal THC induced platelet aggregation, highlighting the potential clinical benefits of antiplatelet therapy in potentially preventing THC induced myocardial ischemia and infarction [43]. Despite this, the current mechanism of 2-AG mediated platelet aggregation remains under investigation as its effects on platelets remain variable, depending on platelet preparation, model under investigation.

In conclusion, there is rising clinical evidence that there are THC dependent effects on myocardial ischemia and infarction. With rising legalization of THC around the US and increasing clinical use it is important to understand the effects of THC exposure. Further studies are needed to understand the interaction between THC, metabolic derangements, inflammation, vasoreactivity, platelet aggregation that result in myocardial ischemia and infarction. Efforts to meticulously characterize patients at risk of suffering from myocardial infarction associated with THC are needed. Additionally, understanding the biochemical pathways involved can help guide therapies. The role of antiplatelets, statins, beta-blockers, calcium channel blockers, antiotensin converting enzyme inhibitor in primary and secondary prevention remains to be seen. Such measure will allow better understanding of the pathophysiology and potential prevention strategies of THC associated myocardial infarction.

## Figures and Tables

**Figure 1:**
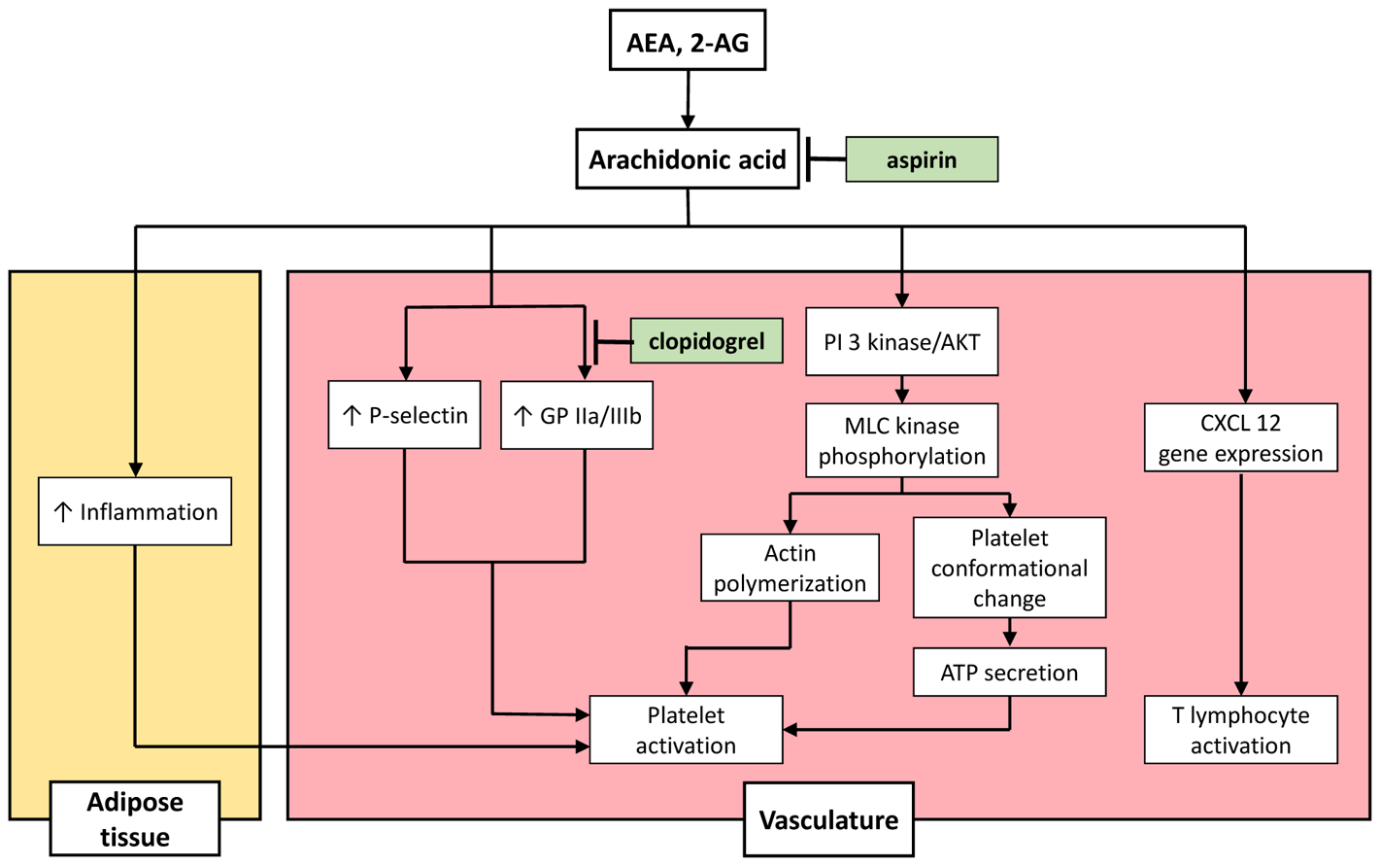
Effect of tetrahydrocannbinoid (THC) from marijuana use and its downstream activation of arachidonyl-ethanolamide (AEA) and 2-Arachidonoylglycerol (2AG) on inflammation and platelet activation at the tissue level. Therapeutic strategies involving aspirin and P2Y12 inhibitor, such as clopidogrel have shown retardation of platelet aggregation in target subjects **Abbreviations:** GP – Glycoprotein, PI 3 kinase – Phosphoinositol-3-kinase, AKT – protein kinase B, MLC – Myosin Light Chain, ATP – Adenosine triphosphate, CXCL –CXC motif chemokine (aka stromal cell-derived factor)”

## References

[1] Staff ALM (2017). Marijuana Legalization Across the U.S.

[2] Terry McElrath YM (2017). Longitudinal patterns of marijuana use across ages 18–50 in a US national sample: A descriptive examination of predictors and health correlates of repeated measures latent class membership. Drug Alcohol Depend.

[3] Tai S, Fantegrossi WE (2017). Pharmacological and Toxicological Effects of Synthetic Cannabinoids and Their Metabolites. Curr Top Behav Neurosci.

[4] U.S Department of Health and Human Services, CDC, NCHS (2017). Underlying Cause of Death 1999–2013 on CDC WONDER Online Database.

[5] Sardarinia M (2016). Risk Factors for Incidence of Cardiovascular Diseases and All-Cause Mortality in a Middle Eastern Population over a Decade Follow-up: Tehran Lipid and Glucose Study. PLoS One.

[6] Jayakody AA (2006). Illicit and traditional drug use among ethnic minority adolescents in East London. Public Health.

[7] Pagotto U (2006). the emerging role of the endocannabinoid system in endocrine regulation and energy balance. Endocr Rev.

[8] Liu J (2012). Hepatic cannabinoid receptor-1 mediates diet-induced insulin resistance via inhibition of insulin signaling and clearance in mice. Gastroenterology.

[9] Racine C (2015). Metabolic Effects of Marijuana Use among Blacks. J Dis Glob Health.

[10] Muniyappa R (2013). Metabolic effects of chronic cannabis smoking. Diabetes Care.

[11] Foltin RW, Fischman MW, Byrne MF (1988). Effects of smoked marijuana on food intake and body weight of humans living in a residential laboratory. Appetite.

[12] Hollister LE, Reaven GM (1974). Delta-9-tetrahydrocannabinol and glucose tolerance. Clin Pharmacol Ther.

[13] Rodondi N (2006). Marijuana use, diet, body mass index, and cardiovascular risk factors (from the CARDIA study). Am J Cardiol.

[14] Vickers SP, Kennett GA (2005). Cannabinoids and the regulation of ingestive behaviour. Curr Drug Targets.

[15] Riggs PK (2012). A pilot study of the effects of cannabis on appetite hormones in HIV-infected adult men. Brain Res.

[16] Smit E, Crespo CJ (2001). Dietary intake and nutritional status of US adult marijuana users: results from the Third National Health and Nutrition Examination Survey. Public Health Nutr.

[17] Mittleman MA (2001). Triggering myocardial infarction by marijuana. Circulation.

[18] Ladeiras LR (2017). The Ratio Between Visceral and Subcutaneous Abdominal Fat Assessed by Computed Tomography Is an Independent Predictor of Mortality and Cardiac Events. Rev Esp Cardiol (Engl Ed).

[19] Schlecht I (2017). Visceral adipose tissue but not subcutaneous adipose tissue is associated with urine and serum metabolites. PLoS One.

[20] Gonzalez N (2017). Regulation of visceral and epicardial adipose tissue for preventing cardiovascular injuries associated to obesity and diabetes. Cardiovasc Diabetol.

[21] Dol Gleizes F (2009). Rimonabant, a selective cannabinoid CB1 receptor antagonist, inhibits atherosclerosis in LDL receptor-deficient mice. Arterioscler Thromb Vasc Biol.

[22] Klein TW, Cabral GA Cannabinoid-induced immune suppression and modulation of antigen-presenting cells. J Neuroimmune Pharmacol.

[23] Ribeiro A (2012). Cannabidiol, a non-psychotropic plant-derived cannabinoid, decreases inflammation in a murine model of acute lung injury: role for the adenosine A(2A) receptor. Eur J Pharmacol.

[24] Burstein S (2015). Cannabidiol (CBD) and its analogs: a review of their effects on inflammation. Bioorg Med Chem.

[25] Ben Shabat S (2006). New cannabidiol derivatives: synthesis, binding to cannabinoid receptor, and evaluation of their antiinflammatory activity. J Med Chem.

[26] Esposito G (2007). Cannabidiol in vivo blunts beta-amyloid induced neuroinflammation by suppressing IL-1beta and iNOS expression. Br J Pharmacol.

[27] Alshaarawy OJ, Anthony C (2015). Cannabis smoking and serum C-reactive protein: a quantile regressions approach based on NHANES 2005–2010. Drug Alcohol Depend.

[28] Ridker PM (2008). Rosuvastatin to prevent vascular events in men and women with elevated C-reactive protein. N Engl J Med.

[29] Liao JK (2009). Rosuvastatin to prevent vascular events in men and women with elevated C-reactive protein. Curr Atheroscler Rep.

[30] Thomas G, Kloner RA, Rezkalla S (2014). Adverse cardiovascular, cerebrovascular, and peripheral vascular effects of marijuana inhalation: what cardiologists need to know. Am J Cardiol.

[31] Rezkalla S, Stankowski R, Kloner RA (2016). Cardiovascular Effects of Marijuana. J Cardiovasc Pharmacol Ther.

[32] Gottschalk LA, Aronow WR, Prakash R (1977). Effect of marijuana and placebo-marijuana smoking on psychological state and on psychophysiological cardiovascular functioning in anginal patients. Biol Psychiatry.

[33] O’Sullivan SE, Kendall DA, Randall MD (2006). Further characterization of the time-dependent vascular effects of delta9-tetrahydrocannabinol. J Pharmacol Exp Ther.

[34] O’Sullivan SE, Kendall DA, Randall MD (2005). vascular effects of delta 9-tetrahydrocannabinol (THC), anandamide and N-arachidonoyldopamine (NADA) in the rat isolated aorta. Eur J Pharmacol.

[35] O’Sullivan SE, Kendall DA, Randall MD (2005). Randall, The effects of Delta9-tetrahydrocannabinol in rat mesenteric vasculature, and its interactions with the endocannabinoid anandamide. Br J Pharmacol.

[36] O’Sullivan SE (2005). Novel time-dependent vascular actions of Delta9-tetrahydrocannabinol mediated by peroxisome proliferator-activated receptor gamma. Biochem Biophys Res Commun.

[37] Barbosa PP (1981). Vasoconstriction induced by delta 9-tetrahydrocannabinol on the perfused rabbit ear artery. Arch Int Pharmacodyn Ther.

[38] White HL, Tansik RL (1980). Effects of delta 9-tetrahydrocannabinol and cannabidiol on phospholipase and other enzymes regulating arachidonate metabolism. Prostaglandins Med.

[39] Ruhaak LR (2011). Evaluation of the cyclooxygenase inhibiting effects of six major cannabinoids isolated from Cannabis sativa. Biol Pharm Bull.

[40] Mach F, Steffens S (2008). The role of the endocannabinoid system in atherosclerosis. J Neuroendocrinol.

[41] Deusch E (2004). The procoagulatory effects of delta-9-tetrahydrocannabinol in human platelets. Anesth Analg.

[42] Signorello MG, Leoncini G (2014). Effect of 2-arachidonoylglycerol on myosin light chain phosphorylation and platelet activation: The role of phosphatidylinositol 3 kinase/AKT pathway. Biochimie.

[43] Keown OP (2010). 2-arachidonyl glycerol activates platelets via conversion to arachidonic acid and not by direct activation of cannabinoid receptors. Br J Clin Pharmacol.

